# Supplementary material for: The pUL47 tegument protein of Marek’s Disease Virus interacts with p32/C1QBP to promote horizontal transmission
Source: PLoS Pathog. 2025 Aug 19;21(8):e1013434. doi: 10.1371/journal.ppat.1013434 (PMC12380274; doi:10.1371/journal.ppat.1013434)
Supplement: Attachment — Submitted filename: Response to Reviewers Comments.docx [file ppat.1013434.s002.docx]

**Additional Editor Comments:**

In the revised version, please, make sure to address all the criticisms. In particular, Reviewer 1 was concerned about the lack of an alternative hypothesis to explain the data that contradict the initial hypothesis and experiments to validate it. Reviewer 2 questioned whether UL47 and p32 interact directly and the role of this interaction in infection.

R: As described in the cover letter, we tried to answer to every point raised by the reviewers, with a particular emphasis on providing an alternate hypothesis for the role of the pUL47-p32 interaction in transmission and a deeper description of the interaction (whether it is direct or mediated by RNA) and its role in infection.

As you can see with reading individual answers or in the cover letter, we provide an alternate hypothesis for the role of the pUL47-p32 interaction in transmission by showing that the interaction domain of pUL47 with p32 is necessary for pUL47 to localize to nuclear speckles during infection (new Figure 7) and for optimal nuclear import (Figure 8). Since pUL47 localizes to the same nuclear speckles as p32 (a co-factor of the ASF-SF2 splicing complex), we suggest that the absence of interaction with p32 results in the absence of localization of mutant pUL47 to splicing-related speckles. In these conditions, mutant pUL47 would be expected to be unable to perform splicing-related functions thus ultimately resulting in the lack of horizontal transmission. This hypothesis is described in lines 328-347 of the revised manuscript.

Although we were unable to formerly demonstrate the pUL47-p32 interaction in infected cells by co-IPs because of technical limitations (please see detailed answer to reviewer #2 point 2), we believe that the results described above also partly answer to reviewer #2’s concerns about the role of the interaction during infection since they provide a functional basis to it. As for reviewer #2’s concern about whether the interaction is direct or mediated by RNA and about the lack of interaction with pUL47Δ71-185, we provided new co-IPs including nuclease assays that show that the interaction is direct (i.e RNA is not necessary) and confirming that pUL47Δ71-185 is unable to interact with p32 in avian cells (new Figure 5C-D).

**Part I - Summary**

 Reviewer #1: In this report by Durand et al., the potential interaction of Marek’s disease virus (MDV) tegument protein UL47 with cellular proteins was investigated. The authors used a yeast two-hybrid system to identify cellular protein p32/C1QBP. The evidence for this interaction is strong, with interactions identified in two different chicken tissue libraries and extensive protein-protein interaction analyses, including co-immunoprecipitation and co-localization in immunofluorescence assays. The authors concluded that the interaction of UL47 and p32 was located within aa 71-185 of UL47. Based on one of the functions of p32 in regulating mRNA splicing and their previous studies investigating UL47’s role in the splicing of MDV UL44 (gC), they hypothesized that the interaction between p32 and UL47 was required for regulating UL44 splicing. However, they never tested whether this interaction affected UL44 splicing in vitro and continued with in vivo studies using a recombinant virus in which the proposed interacting residues on UL47 (71-185) were deleted. They found no difference in the localization of UL47 in cell culture and no difference in replication in experimentally infected chickens compared to wild-type MDV. However, the deletion mutant was unable to transmit to chickens, similar to their previous studies in which the complete UL47 gene was deleted. Analysis of UL44 mRNA splicing in feathers and skin revealed no differences between the MDV wild-type and the deletion mutant, disproving their hypothesis. The manuscript's strengths include the convincing interaction data, the sound in vivo experiments, and, particularly, the use of a natural virus-host in vivo model system to test their hypothesis. The significant weaknesses include the oversimplified interpretation of the results, and further characterization and experiments are needed to address their in vivo results.

Reviewer #2: Durand et al. characterize a distinct domain in the MDV tegument protein, UL47, which is essential for horizontal transmission in its native host (chickens). The data demonstrating an absence of horizontal transmission in their UL47 del71-185 mutant virus as compared to wild type is very convincing and poses an interesting model for decoupling replication and transmission. We would ask the authors to strengthen their conclusions regarding the interaction between UL47 and p32. In particular, there is an absence of data demonstrating the UL47-p32 interaction in the context of MDV infection. There is also no data directly connecting the lack of p32 interaction to the absence of horizontal transmission. We find the article is well written, although improvements could be made to the figures and legends for readability.

Reviewer #3: In this manuscript, the authors investigated how the pUL47 tegument protein contributes to Marek’s disease virus transmission. They used yeast-two hybrid screens to identify interaction partners of pUL47. They identified the cellular p32 (C1QBP) protein and mapped the interaction domain in pUL47. Deletion of this interaction domain did not affect other functions of pUL47 or pathogenesis of the respective mutant virus; however, virus shedding was completely abrogated in a gC independent manner. This study is well written and contains exciting data for the field and beyond, as the role of pUL47 in virus transmission can only be assess in very few natural virus/host models. The authors should address the following points prior to publication.

**Part II – Major Issues**

Reviewer #1: 1. The overall subject of the paper involves the interaction between UL47 and p32, which is believed to be crucial for regulating mRNA splicing. However, their data completely refutes this hypothesis, which is not a problem per se, but, alternative mechanisms are not addressed. For example, the authors state that removing aa 71-185 does not affect UL47 localization in vitro and assume this does not affect its overall function. However, is a negative result. It has been published that localization of MDV UL47 is nuclear in cell culture and nucleocytoplasmic in skin cells in chickens. Similar results are seen for HSV UL47 in vitro. The authors should address the localization of UL47 (del 71-185) in skin cells to fully conclude that localization is not affected by this deletion. Alphaherpesvirus UL47 proteins are highly complex, and their localization in cells is tightly regulated, at least in the context of HSV studies. Thus, the authors cannot conclude localization has not been affected using in vitro studies, as the protein is not localized normally in cell culture. It is possible that this region is crucial for the shuttling of UL47 from the nucleus to the cytoplasm in vivo that would likely be important for its function.

R: We thank the reviewer for the suggestion regarding alternative explanations as to UL47Δ71-185’s phenotypes, which we investigated as detailed below.

We agree that *in vitro* studies cannot replace *in vivo* studies in terms of biological relevance. However, the large majority of studies regarding cellular localization of proteins rely on *in vitro* results and their conclusions are usually assumed to apply *in vivo* for obvious technical reasons, yet their relevance is rarely questioned.

The description of the localization of pUL47 in infected skin cells *in vivo* is an exception: only one laboratory successfully managed and described the cytoplasmic localization of MDV pUL47 in FFE cells in chickens, but they showed only two/three cells and at low magnification (Jarosinski et al., JVI2012). To reproduce such an experiment would have required extensive time and resources that we believe were beyond the scope of the revision of this manuscript.

The reviewer states that “Similar results [i.e that HSV UL47 is nucleocytoplasmic in skin cells as opposed to nuclear in cell culture] are seen for HSV UL47” and “the authors cannot conclude localization has not been affected using in vitro studies, as the protein is not localized normally in cell culture”. Unless we are mistaken, the *in vivo* localization of HSV of pUL47 is unknown, so there is no way of knowing whether the conclusions made on HSV pUL47 in cell culture are representative or “normal” of what happens *in vivo*. In addition, we described the exact same localization for MDV pUL47 as for HSV pUL47 in cell culture. Therefore, in our opinion, pUL47 HSV and pUL47 MDV are very similar *in vitro* (and both interact with p32), the only difference being that some evidence of a differing localization exists *in vivo* for MDV in skin cells but not for HSV (which could actually have similar features).

However, following the reviewer’s recommendations, we investigated MDV pUL47’s nucleocytoplasmic shuttling properties. We constructed a virus with the UL47Δ71-185 mutation together with a C-terminal GFP fusion to pUL47 (vUL47Δ71-185-GFP), which allowed us to monitor the localization of mutated pUL47 in the context of infection (in cell culture). We observed that the mutated protein is less nuclear and, importantly, that it lacks punctate localization, as opposed to WT pUL47 where the protein is mostly nuclear and shows nuclear punctates as observed in transfected cells (Figure 7). This shows that the deletion can be associated to a modified localization of the protein in infected cells.

Additionally, we compared the shuttling properties of pUL47 WT and pUL47Δ71-185 in infected cells as suggested by the reviewer, either using leptomycin-B (LMB) or using FLIP (Fluorescence Loss in Photobleaching) in living cells. LMB experiments showed that both pUL47 WT and pUL47Δ71-185 are capable of nuclear export as both are sensitive to LMB (Figure 7 and lines 214-229). FLIP experiments show that both pUL47 WT and pUL47Δ71-185 are capable of nuclear import although pUL47Δ71-185 nuclear import is delayed compared to pUL47 WT (Figure 8 and lines 230-245)

These observations show that in infected cells MDV pUL47 is indeed capable of shuttling like HSV pUL47, but that its punctate localization seems to be associated to its capacity to bind nuclear p32 (please see our response to point #3 regarding p32 localization). We discuss the consequences of these results on global interpretation in lines 332-349.

2. In addition to the localization results, the removal of this region of UL47 is likely to affect multiple aspects of the protein's function. The results show that mRNA splicing was unaffected by the removal of these sequences; however, transmission was completely abrogated. What else is present in this region that could interfere with UL47 function in skin cells? Arginine rich regions can have many important functions that are not addressed here. Further studies, or at least hypotheses, should be developed and addressed. It would also be worthwhile to define the interaction of UL47 with p32 better and mutate small sequences rather than removing large portions of the protein that likely affect multiple aspects of UL47’s function.

R: We did try to define a smaller interaction domain. As shown in Figure 4, we tested domains 1-71, 71-119 and 119-185 (lanes 9-11 in Figure 4A of the original manuscript) and those failed to show any interaction with p32(M9) in Y2H assays, thus showing that the minimal domain was 71-185. In the same figure, we also mutated serine-arginine (SR) domains (including multiple domains) to check for interaction with p32(M9) (Figure 4B) and these mutations had only mild effects in Y2H assays (Figure 4C).

These results were not very well detailed in the original manuscript, so we highlighted them in lines 150-151 and lines 166-168 to better justify the use of domain 71-185 thereafter. In addition, we modified Figure 4 to make it more readable, as suggested by reviewer #3.

3. The results in p32 localization and UL47 interactions is confusing. The localization of p32 (M9, D30 truncated and epitope-tagged) and cellular p32 is conflicting and has not been fully addressed. The results suggest that these do not identify the same protein. Figure 3D is not convincing. The full p32 cDNA should be cloned, and its localization and interaction with UL47 should be addressed. The current results are not well supported and raise more questions for this study.

R: We agree that localization of p32 in its different forms is confusing. We did clone FL chicken p32 (associated to a Cterminal GFP tag) but its expression is essentially cytoplasmic and mitochondrial, because the mitochondrial target sequence (MTS) is hardly cleaved, as observed with endogenous p32. As a result, since the interaction is expected to take place in the nucleus, we anticipate that co-IP with pUL47 would fail by lack of sufficient amounts of nuclear p32.

In the original manuscript, we mentioned that human p32 can be cleaved after the MTS at residue 73 (lines 106-107 and arrow in Figure 1A). Since p32 is a known regulator of splicing factor ASF/SF2 (line 115 of the original manuscript), we know that the mature, nuclear form exists. Importantly, we noted that, based on sequence alignment, the putative cleavage site is very close to the beginning of the M9 sequence (lines 111-112). We thus supposed that p32(M9) represents a constitutively “mature” form of p32 and that this form, which lacks the MTS and is therefore unable to be targeted to mitochondria, is mostly nuclear as we indeed observed. In the manuscript, we concentrated on this form (which our commercial antibody cannot detect in fixed cells as shown in Figure 3D) because it made the most sense regarding what is known on pUL47 localization and functions. The endogenous nuclear form of p32 is not abundant enough to be detected by IF, at least not with our antibody. We discussed alternate possibilities which included the role of mitochondrial p32 (lines 321-324 of the original manuscript) during infection.

In an attempt to better explain the focus on p32(M9), we highlighted the role of the MTS sequence (lines 109-110) and that p32(M9) is expected not to localize to mitochondria after cleavage (lines 115-116). However, we feel that adding the p32(FL) overexpression data (including WB and IF characterization as well as IP with pUL47) would add to the confusion between the different forms of p32 that the reviewer mentioned.

Reviewer #2: 1. UL47 is an RNA binding protein, as demonstrated by prior studies with MDV and HSV1. This makes it probable that the interaction between UL47 and p32 is mediated by RNA. The authors should repeat their Co-IP experiments with benozonase (or a comparable nuclease) to evaluate if the binding is lost after nuclease treatment. The current data cannot conclude whether the UL47-p32 interaction is direct (protein-protein) or indirect (protein-RNA-protein).

R: We thank the reviewer for this suggestion. We repeated the co-IP experiments in triplicate with or without micrococcus nuclease, as suggested (Figure 5C of the revised manuscript). We carried out this experiment also with pUL47Δ71-185 to answer to the reviewer’s third major point (see below). Results showed that the interaction between pUL47 and p32(M9) is unaffected by the absence of nucleic acid. Analysis of the RNA present in the lysate showed that it was degraded, independently of the nuclease treatment. Indeed, we could recover RNA in only one experiment out of three and analysis of this RNA showed degradation when compared to control RNA (Figure 5D). This is not surprising as the lysis protocol is quite extensive and exposes RNA to cellular nucleases for a long time. Of note, for these three new experiments and contrary to the original experiments, we did not include EDTA in the first lysis step so that the added nuclease would not be blocked. Results have been added in lines 182-188 of the revised manuscript.

2. Currently there is minimal data supporting UL47-p32 interaction or colocalization in the context of infection. Almost, if not all, of the UL47-p32 interaction findings were made via ectopic expression of labeled constructs. Only one figure panel (Fig. 3C) used an actual virus and this panel did not recapitulate the findings of their expression constructs (likely due to antibody limitations). The authors should provide additional data to support the existence of the UL47-p32 interaction in the context of infection. I’m curious if the nuclear speckles persist during an actual infection, or if these seed proper replication compartments. If possible, a time course examining UL47-p32 interactions during infection would be helpful in clarifying the functional outcome of this interaction.

R: We agree that data in infected cells were lacking. The reason why over-expression of tagged proteins is preferred for co-IPs is that strong expression and good detection of tested proteins is important for successful detection of interaction, and natural expression of viral or cellular proteins is often insufficient. We nevertheless constructed a virus with the UL47Δ71-185 mutation together with a C-terminal GFP fusion to pUL47 (vUL47Δ71-185-GFP), which allowed us to compare the localization of mutated and WT pUL47 in the context of infection to answer reviewers #1 and #2’s concerns. We used this virus and vUL47GFP to immunoprecipitate pUL47GFP from infected cells and look for endogenous p32 in the IP using our commercial antibody.

The picture below shows the results from one out of three IPs: the WB on the left shows probing with the anti-p32 antibody and the WB on the right is the same blot but reprobed with an anti-GFP. As shown in the top-right gel, pUL47GFP is strongly degraded in lysates from infected cells despite the use of anti-proteases and carrying out the experiment at 4°C (arrows indicate the expected full-length forms, essentially visible in the pre-immunoprecipitation inputs). In addition, immunoprecipitation efficiency of full-length pUL47GFP is poor (as shown in the right-bottom gel), probably because the protein is already involved in viral complexes. As a result of degradation and poor IP efficiency of UL47GFP, we could not observe any specific co-precipitation of endogenous p32 (bottom-left gel).

We considered using PLA in infected cells, but the fact that our p32 antibody cannot detect the nuclear form of p32 combined with poor cytoplasmic p32 levels in infected cells (see Figure 7 for instance) made this technique unappealing.

Given these experimental limitations, we cannot experimentally confirm that the p32/pUL47 interaction also exists in infected cells. However, the interaction was characterized by using two independent and different techniques (Y2H and co-IP) and was also identified by another team, on another herpesvirus (HSV-1) by another technique (pull-down) (Liu et al., JVI 2015) and we therefore believe it to be solid and plausible during infection. Nevertheless, we have acknowledged the lack of a former demonstration of the interaction in infected cells in the discussion (line 329) of the revised manuscript.

As for the relation between pUL47’ speckles and replication compartments, we included a VP5 staining of cells infected with vUL47-GFP or vUL47Δ71-185-GFP in Figure 7 showing that pUL47’ speckles are distinct from replication compartments. This point is described in the text in line 223-225.

3. We would ask the authors to strengthen their data supporting a lack of interaction between UL47 del71-185 and p32 (Fig. 5). In Fig. 5C the decrease in V5 levels after myc IP for the del71-185 mutant appears similar to the decrease in total UL47 expression for the mutant. I’m also uncertain which protein band is p32 in the V5 panel of Fig 5C (myc IP), as there are two bands present. This doublet was not present in prior p32 blot images, which leads me to question if it’s been cropped or is a new band. We would ask the authors to provide additional replicates (with more comparable expression of UL47 constructs) or an alternative method to demonstrate UL47 del 71-185 fails to interact with p32 (e.g. PLA immunofluorescence). This is particularly necessary as the IF in Fig. 5B demonstrates colocalization of UL47 del 71-185 with p32.

R: The doublet the reviewer refers to actually consists on an upper band of immunoglobulins (from the antibodies used for IP) and a lower band that corresponds to p32, we apologize for the confusion. Igs are not visible on the new WB (see below).

To answer to the reviewer’s concerns about differing amounts of expression of pUL47wt and pUL47Δ71-185 and their consequence on the IP levels of p32, we repeated the experiment with optimizing the relative quantities of plasmids used for transfection. As shown in Figure 5C, pUL47Δ71-185 still failed to immunoprecipitate p32 despite quantities similar to those of pUL47wt and despite lower amounts of p32 in lysates from cells expressing pUL47wt than in cells expressing pUL47Δ71-185.

We would like to emphasize that colocalization of pUL47Δ71-185 with p32 is not necessarily associated with direct interaction. Instead, it shows that localization of the mutant protein is not directly related to p32 (in transfected cells) and is not impaired by the deletion, which we emphasized as a positive sign that the deletion is not deleterious in the original manuscript (lines 180-181 and 270).

This is particularly important because additional data show that, contrary to pUL47 WT, pUL47Δ71-185 does not localize to nuclear punctates in infected cells (Figure 7 and lines 214-229 of the revised manuscript). Our interpretation is that pUL47 localizes to RNA-related nuclear punctates independently of p32 (but the interaction is possible if domain 71-185 is present) and that in infected cells, the protein is exported if it is not retained there by the 71-185 domain, presumably through its interaction with p32. This hypothesis is also based on data described in Figure 7, suggesting that nuclear export of pUL47Δ71-185 is stronger than pUL47 WT (Figure 7) whereas nuclear import is less efficient (Figure 8 and lines 230-245).

**Part III – Minor Issues: Editorial and Data Presentation Modifications**

Reviewer #1: 1) The authors utilize viral genomes as evidence of viral shedding, a standard method for analyzing MDV transmission. They found little difference in genomes shed but never addressed whether infectious virus is shed between the two viruses. Thus, it appears that the birds are shedding the virus; so, where is the defect in transmission? Further discussion of this should be explained.

R: This intriguing phenotype was already observed and discussed with a virus depleted for UL47 (see Chuard et al., JVI2020) but it was not in this manuscript. Therefore, we modified the introduction (lines 83-86) and added the following statement to the discussion to make the working hypothesis for the role of pUL47 in entry clearer: “A likely explanation for this phenotype is that pUL47 performs one or several early functions during entry in the host that are essential to initiate infection.” (lines 306-308).

Reviewer #2: 1. Remove the text “suggest that pUL47 may contribute to reprogramming cellular splicing to promote transmission” from the Abstract. This is speculative and best suited to the discussion.

R: This sentence has been removed.

2. Generally data presentation and transparency should be improved including the following:
a. Label western blots with size markers (Fig. 1C-D, 5C-D). R:Done.
b. Clearly indicate the number of replicates for each panel, for n≤5 data points should be present in the graphs (Fig. 4C, 7)

R: We have included the data points in figure 4C (where n=3) and the number of replicates in the figure legend. We did not include the data points in Figure 7 because those are >5 (up to 9 points for some conditions).

c. In panel 6C how many plaques were measured & how many biological replicates did this correspond to.

R: We have indicated the number of plaques measured per condition (50) and the number of biological replicates (1) in the figure legend. We did not include more biological replicates because deletion of the complete UL47 gene did not impact plaque size (Chuard et al. JVI2020) as stated in line 198-199 of the original manuscript, so deletion of domain 71-185 was expected to have no effect, as observed.

3. Figure legends could have increased clarity including the following:
a. Statistical test used and what conditions were compared. The legend should detail what p-value range the asterisk denotes (Fig. 4C, 6C, 7C-D)

R: We have added this information for figure 4C as it was indeed missing. However, it was already indicated in the original manuscript for figures 6C (lines 895-897) and 7 (lines 909-912).

b. Indicate either the cell type or source material (animal vs. cell culture) assessed in the assay. For instance Fig. 2 , 3, 5 just says “Cells were transfected”. Based on the methods section I’m guessing they used CESCs, but it would be preferable if they said CESC instead of cells.

R: We apologize for the confusion. All cell types are now specified in the legends and the use of ESCDL-1 cells for transient expression assays is now also specified in Methods (lines 523-524).

Reviewer #3: 1) The authors make the exciting observation that virus transmission is abrogate upon deletion of UL47 aa71-185 in vivo. Their data clearly shows that the UL47Δaa71-185 virus is still fully pathogenic, but that transmission is completely abrogated. However, they also show that shedding of this virus occurs efficiently, as high virus levels were detected in the dust. This suggests that either the shed UL47Δaa71-185 virus is defective or that it can not enter and/or infect the target cell in the lung. As addressing these questions is beyond the scope of this manuscript, the authors should at least extensively discuss these options in the Discussion section.

R: This intriguing phenotype was already observed and discussed with a virus depleted for UL47 (see Chuard et al., JVI2020) but it was not in this manuscript. Therefore, we modified the introduction (lines 83-86) and added the following statement to the discussion to make the working hypothesis for the role of pUL47 in entry clearer: “A likely explanation for this phenotype is that pUL47 performs one or several early functions during entry in the host that are essential to initiate infection.” (lines 306-308).

2) Viruses do not “survive” (e.g. line 56 “key part of viruses’ life cycle for survival in the host population.”). They spread and/or are maintained in a population. Please rephrase this. R: We replaced the entire sentence by “Host-to-host transmission is essential for viruses to be effectively maintained in the host population” in the revised manuscript.
3) Line 64ff: correct to “alphaherpesviruses” R: Corrected together with two other occurrences in the Discussion.
4) Line 66: The authors state that the MDV “genome is integrated to the cellular genome during latency”. The virus specifically integrates into host telomeres. Please correct his and provide a reference for your statement. R: We amended the sentence as suggested and added the review by Kheimar et al. as well as two papers describing experimentally the integration as reference.
5) Line 70: “excretion from the FFE”. Excretion has not been formally shown to my knowledge. Maybe use “release” instead. R: Thanks for the suggestion, modification done.
6) The fond size in Table 1 is two small. Please optimize.

R: We were requested to include the Table in the core of the text, which resulted in very small font size, we apologize for this.

7) Figure 4B: please properly align the labels right and left of the UL47 overview (especially for 19-21). R: All labeling for this figure has now been updated for better readability.
8) Line 164ff: “Invalidate” does not fit in this context (e.g. “does not invalidate functions of pUL47”). Abrogate or block would fit better. R: Thanks for the suggestion, we changed for “abrogate”.
9) Figure 8B. Please indicate the full length, 104 and 145 on the right as nicely done for Figure 8A.R:Done.
10) Line 282: please properly cite the Liu et al. paper. R :Done.
